# Breath analysis by two-dimensional gas chromatography with dual flame ionisation and mass spectrometric detection – Method optimisation and integration within a large-scale clinical study

**DOI:** 10.1016/j.chroma.2019.02.001

**Published:** 2019-06-07

**Authors:** Michael J. Wilde, Rebecca L. Cordell, Dahlia Salman, Bo Zhao, Wadah Ibrahim, Luke Bryant, Dorota Ruszkiewicz, Amisha Singapuri, Robert C. Free, Erol A. Gaillard, Caroline Beardsmore, C.L. Paul Thomas, Chris E. Brightling, Salman Siddiqui, Paul S. Monks

**Affiliations:** a Department of Chemistry, University of Leicester, University Road, Leicester, LE1 7RH, UK; b Centre of Analytical Science, Loughborough University, Epinal Way, Loughborough, LE11 3TU, UK; c Leicester NIHR Biomedical Research Centre (Respiratory theme), Glenfield Hospital, Groby Road, Leicester, LE3 9QP, UK; d College of Life Sciences, Department of Infection, Immunity and Inflammation, University of Leicester, University Road, Leicester, LE1 7RH, UK; e Paediatric Clinical Investigation Centre, Leicester NIHR Biomedical Research Centre (Respiratory theme), University of Leicester, Leicester Royal Infirmary, Leicester, LE2 7LX, UK

**Keywords:** GC×GC, Comprehensive two-dimensional gas chromatography, Flow modulation, Breath analysis, Metabolomics, Precision medicine

## Abstract

Precision medicine has spurred new innovations in molecular pathology leading to recent advances in the analysis of exhaled breath as a non-invasive diagnostic tool. Volatile organic compounds (VOCs) detected in exhaled breath have the potential to reveal a wealth of chemical and metabolomic information. This study describes the development of a method for the analysis of breath, based on automated thermal desorption (TD) combined with flow modulated comprehensive two-dimensional gas chromatography (GC×GC) with dual flame ionisation and quadrupole mass spectrometric detection (FID and qMS). The constrained optimisation and analytical protocol was designed to meet the practical demands of a large-scale multi-site clinical study, while maintaining analytical rigour to produce high fidelity data. The results demonstrate a comprehensive method optimisation for the collection and analysis of breath VOCs by GC×GC, integral to the standardisation and integration of breath analysis within large clinical studies.

## Highlights

• New method for the analysis of exhaled breath VOCs by TD-GC × GC-FID/qMS.
• Optimisation of flow modulation and dual detection alongside clinical requirements.
• Addresses key challenges of using GC × GC for large-scale breath metabolomics.

## 1 Introduction

Comprehensive two-dimensional gas chromatography (GC×GC) is an advanced analytical technique used for the analysis of complex organic matrices; its main advantage is the unparalleled separation power it can afford over conventional one-dimensional gas chromatography. However, for many applications, one-dimensional gas chromatography combined with mass spectrometric or flame ionisation detection (GC–MS and GC-FID) is still considered the ‘gold standard’ [1], despite the potential benefits realised by GC×GC. GC×GC has been utilised for a wide range of applications, with increasing application in the detection of biomarkers within metabolomics studies [[2], [3], [4]]. However, the majority are performed on a small scale and involve the use of expensive or specialist detectors and modulators, resulting in high consumable and technical costs. In addition, GC×GC produces extensive data-rich chromatograms which require specialist knowledge to interpret.

Breath analysis is rapidly developing as a new branch of molecular pathology and metabolomics and is known as ‘breathomics’. Small molecular-weight metabolites from metabolic processes occurring within the body are released into the bloodstream, transferred across the alveolar membrane and are exhaled in breath as volatile organic compounds (VOCs). Molecular characterisation of these exhaled VOCs has shown potential in aiding medical diagnosis [5]. Several breath tests have received EU and FDA approval [6], with more being developed for a range of diseases such as the diagnosis of tuberculosis [7], inflammatory bowel disease [8] and liver disease [9].

Large-scale, multi-site clinical studies are beginning to integrate breath analysis as part of their protocols, demonstrating the translation of laboratory benchtop techniques, developed in proof-of-concept studies, to clinical bedside technologies. The method described herein was optimised and integrated as part of a large clinical study, within the East Midlands Breathomics Pathology Node (EMBER), for the discovery of novel breath biomarkers to stratify patients with self-reported acute breathlessness [10].

Common technologies employed for the discovery analysis of breath include offline, online and real-time ion mobility (IMS) and mass spectrometric techniques. These include proton transfer reaction- and select ion flow tube-mass spectrometry (PTR-MS and SIFT-MS) [11,12], GC-IMS [13] and thermal desorption GC–MS, which is generally accepted as the benchmark technique for the discovery of breath biomarkers [14]. Previous research, albeit sparse, has demonstrated the potential of GC×GC for breath analysis, with the number of VOCs detected exceeding those detected by conventional GC–MS [15,16]. This has generated interest within the breath research community, however, most studies were conducted on a small scale, including no more than 60 patients and contained little information or figures demonstrating the chromatographic separation, optimisation of the method and its integration as part of a large clinical study.

The objective of this work was to report the development of a robust GC×GC method for the analysis of exhaled breath VOCs and demonstrate its optimisation alongside an end-to-end quality controlled sample pathway, as part of a routine method integrated within a large clinical study. The current work provides a detailed assessment of the chromatographic optimisation as well as an objective evaluation of the method when integrated within a clinical protocol. The method provides increased chromatographic separation compared with GC–MS, while the choice of a thermal desorption autosampler increases sample throughput, and the lower cost and easy to use flow modulator and quadrupole mass spectrometer moves towards making the advanced analytical technique more accessible for significant sample volumes.

## 2 Material and methods

### 2.1 Reagents and materials

A reference solution used to monitor retention behavior was prepared from a 40 mg/L C_8_-C_20_ saturated alkanes certified reference material (Sigma Aldrich, Dorset, UK) combined with a 2000 μg/mL aromatics calibration standard (NJDEP EPH 10/08 Rev.2, Thames Restek, Saunderton, UK). The mixture was diluted in methanol (SupraSolv grade, Sigma Aldrich, Dorset, UK) to give final concentrations of 20 μg/mL and 10 μg/ml for n-alkanes and aromatics, respectively (Table S1). Two performance mixtures were prepared, a 100 μg/mL multi-component indoor air standard (Sigma Aldrich, Dorset, UK) and a 280–540 μg/mL multi-component GC programmed test mix (Sigma Aldrich, Dorset, UK) were diluted individually in methanol to give final concentrations of 10 μg/mL and 7–14 μg/mL, respectively (Tables 1 and S2). An internal standard solution was prepared from 2000 μg/mL toluene-d_8_ and phenanthrene-d_10_ certified reference solutions (Sigma Aldrich, Dorset, UK) and n-octane-d_18_ (D, 99% Cambridge Isotope Laboratories, Tewksbury, US). The deuterated materials were combined and diluted in methanol to give a final concentration of 20 μg/mL per analyte.

**Table 1:** Summary of retention times and GC×GC performance for 40 components within the indoor air mixture (n = 6), including average and RSD for 1^st^ dimension and 2^nd^ dimension retention time (^1^t_R_ and ^2^t_R_), average 1^st^ and 2^nd^ dimension peak widths (${\bar{w}}_{b}$), average 1^st^ and 2^nd^ dimension peak symmetries, average modulation ratio (M_R_), average number of FID and qMS scans/modulated peak.

	^1^t_R_ (min)		^2^t_R_ (s)		${\bar{w}}_{b}$(s)		Symmetry		M_R_	Scans	
Compound	Mean	RSD %	Mean	RSD %	^1^D	^2^D	^1^D	^2^D		FID	MS
isopropyl alcohol	7.6	0.3	2.2	0.3	15.0	0.36	2.3	1.3	5.0	36	15
acetone	8.0	0.0	0.9	0.7	13.0	0.46	1.9	1.5	4.3	46	11
2-butanone	10.2	0.2	1.2	0.3	18.5	0.30	1.7	1.2	6.2	30	12
hexane	10.3	0.2	0.2	2.7	11.5	0.20	1.6	1.3	3.8	20	7
ethyl acetate	10.8	0.3	1.1	1.0	19.0	0.31	2.5	1.4	6.3	31	10
2,4-dimethyl pentane	11.3	0.2	0.2	2.5	15.0	0.24	1.7	1.4	5.0	24	7
benzene	12.7	0.2	1.3	0.4	15.0	0.28	2.1	1.3	5.0	28	8
2,2,4-trimethyl pentane	13.9	0.1	0.2	1.9	11.5	0.21	1.4	1.3	3.8	21	5
heptane	14.5	0.0	0.2	1.2	10.5	0.20	1.3	1.5	3.5	20	6
methyl isobutyl ketone	16.6	0.0	1.3	0.5	15.0	0.27	2.3	1.3	5.0	27	8
toluene-D8	18.0	0.0	1.4	0.3	12.5	0.27	1.6	1.4	4.2	27	9
toluene	18.2	0.2	1.4	0.8	16.5	0.28	2.0	1.3	5.5	28	8
octane-d18	19.5	0.0	0.3	1.5	12.0	0.20	1.7	1.4	4.0	20	5
octane	20.2	0.0	0.3	1.4	9.5	0.20	1.1	1.5	3.2	20	5
acetic acid butyl ester	21.0	0.0	1.2	0.6	18.0	0.28	3.0	1.4	6.0	28	8
ethylbenzene	23.9	0.1	1.1	0.8	12.5	0.26	1.8	1.5	4.2	26	8
m/p-xylene	24.4	0.1	1.1	0.9	16.5	0.29	1.4	1.5	5.5	29	8
styrene	25.6	0.0	1.7	0.2	13.0	0.29	1.9	1.3	4.3	29	10
o-xylene	25.7	0.0	1.2	0.4	12.0	0.25	1.7	1.2	4.0	25	7
nonane	26.0	0.1	0.2	2.0	10.5	0.19	1.2	1.3	3.5	19	5
α-pinene	27.9	0.1	0.4	1.8	10.5	0.19	1.3	1.3	3.5	19	5
3-ethyltoluene	29.2	0.0	0.8	0.7	9.5	0.22	1.1	1.4	3.2	22	6
4-ethyltoluene	29.3	0.0	0.8	0.5	10.0	0.21	1.0	1.4	3.3	21	6
benzene, 1,3,5-trimethyl-	29.5	0.1	0.8	1.0	9.5	0.24	1.1	1.4	3.2	24	6
2-ethyltoluene	30.0	0.1	0.9	1.0	10.0	0.24	1.0	1.5	3.3	24	7
β-pinene	30.1	0.0	0.4	0.8	9.0	0.18	1.0	1.3	3.0	18	5
1,2,4-trimethyl benzene	30.7	0.0	0.9	0.7	11.0	0.21	1.4	1.4	3.7	21	6
decane	30.8	0.0	0.2	1.5	9.0	0.17	1.0	1.2	3.0	17	5
1,2,3-trimethyl benzene	31.9	0.1	0.9	0.7	9.5	0.24	1.1	1.5	3.2	24	6
(R)-(+)-limonene	32.3	0.0	0.4	0.7	9.0	0.18	1.0	1.4	3.0	18	6
undecane	34.9	0.0	0.1	1.7	9.0	0.17	1.0	1.4	3.0	17	4
nonanal	35.1	0.0	0.7	1.1	12.0	0.21	1.7	1.4	4.0	21	7
1,2,3,4-tetramethyl benzene	35.7	0.0	0.8	0.5	9.0	0.21	1.0	1.4	3.0	21	6
dodecane	38.4	0.0	0.1	1.8	9.0	0.17	1.0	1.3	3.0	17	5
decanal	38.6	0.0	0.6	0.9	10.5	0.21	1.3	1.6	3.5	21	5
tridecane	41.5	0.0	0.1	4.4	8.5	0.17	0.8	1.5	2.8	17	5
tetradecane	44.4	0.1	0.1	3.9	7.5	0.17	0.4	1.3	2.5	17	5
pentadecane	47.1	0.0	0.1	4.6	8.5	0.17	0.8	1.5	2.8	17	5
hexadecane	49.7	0.0	0.1	1.8	9.0	0.16	1.0	1.3	3.0	16	4
phenanthrene-D10	55.1	0.2	2.8	0.6	22.5	0.34	2.7	0.9	7.5	34	8

To aid with the assignment of chemical identities of the VOCs detected in breath and evaluate the separation of various VOCs and chemical classes under the optimised chromatographic method, additional reference mixtures were analysed (Table S3). These included a 100 μg/mL terpene mixture (Spex Centriprep component terpene kit, Emerald Scientific, San Luis Obispo, US), a 57–62 μg/mL alcohol and chloroalkane mixture and a 10 μg/mL menthyl mixture (Sigma Aldrich, Dorset, UK).

### 2.2 Study and ethics

Within EMBER, a prospective real world observational study was carried out involving adults and children presenting with self-reported acute breathlessness across three acute admissions units in Leicester, UK [10]. Written informed consent was obtained from all participants. The study protocol was approved by the National Research Ethics Service Committee East Midlands (REC number: 16/LO/1747) IRAS 198921.

### 2.3 Sample collection

For each patient, online spectrometric data, clinical pathology samples and multiple sorbent tubes containing exhaled VOCs were collected in-clinic. Online in-clinic breath measurements included breath analysis by PTR-TOF-MS, GC-IMS and atmospheric pressure chemical ionisation-mass spectrometry (APCI-MS). The sorbent tubes were sent to Loughborough University and the University of Leicester for analysis by TD-GC-MS and TD-GC×GC-FID/qMS, respectively [10].

Breath samples were collected using the ReCIVA breath sampler device (Owlstone Ltd, Cambridge) [17]. The device comprised of a handheld unit; a disposable face mask; up to four sorbent tubes held within four ports located above two pumps and pressure sensors (Fig. S1). The pressure sensors in the mask and ports monitored the patient’s breathing rate and breath profile. This allowed the software to activate the pumps on and off at specific phases of the breath cycle e.g. to capture the breath during early or late expiration. When the pumps turned on, exhaled breath from the face mask was drawn through the sorbent tubes trapping and pre-concentrating the exhaled VOCs. The gated collection and controlled flow of breath through the tubes allowed the operator to control the volume and portion of breath collected. An air supply, comprised of a pump, tubing and cylinder of activated carbon, delivered purified air to the mask at >30 L/min.

The ReCIVA was set to collect 1 L of exhaled breath from the lower airways using the predefined breath sections in the software, at a flow rate of 250 mL/min. The multi-bed sorbent tubes contained Tenax/TA with Carbograph 1TD (Hydrophobic, Markes International Ltd). As only the back two ports of the ReCIVA were used with sorbent tubes during one collection cycle, solid aluminum tubes were positioned in the front two ports.

At the time of breath sampling, a room air sample and an air supply sample were also collected. Room air sampling involved attaching a sorbent tube to the inlet of a handheld air sampling pump (Escort® Elf, Sigma Aldrich, Dorset, UK). The pump was set to draw room air through the sorbent tube at 0.5 L/min for 2 min collecting 1 L in total. An air supply sample was collected every morning and afternoon when a breath sample was collected. A stainless steel T-piece was connected to the end of the air supply of the ReCIVA to allow the excess air flow to vent while a sorbent tube was attached to the T-piece with the handheld pump connected to other end of the tube. The pump was turned on, sampling the air supply at 0.5 L/min for 2 min collecting 1 L in total. The room air and air supply samples were stored, prepared and analysed using the same analytical procedure as the breath samples.

### 2.4 Sample storage and preparation

After sampling, the sorbent tubes were immediately capped (brass caps, Markes International Ltd, Llantrisant, UK) and placed in a fridge at 4 °C until ready for collection or dispatch to the laboratory, typically within 72 h. To improve sample stability, the samples were dry purged on arrival for 2 min using nitrogen (CP grade with an in-line trap; BOC, Leicester, UK) at a flow rate of 50 mL/min and then stored in the fridge at 2 °C until ready for analysis. Dry purging with a dry clean inert gas reduces the condensed water content from the tube prior to thermal desorption allowing low split or splitless injections during thermal desorption [18]. The low flow rate avoids reaching the breakthrough volume of the VOCs.

Before analysis, the samples were loaded with internal standard solution using the calibration solution loading rig (CSLR, Markes International Ltd, Llantrisant, UK). A 0.6 μL aliquot of internal standard solution was injected onto the tube in a stream of nitrogen at a flow rate of 100 mL/min for 2 min, purging the excess solvent. The n-alkane and aromatic solution and performance mixtures were loaded onto blank tubes using the same method, using a 1 μL aliquot of n-alkane and aromatic solution.

### 2.5 TD-GC×GC-FID/qMS

Analysis by two-dimensional gas chromatography was conducted using an Agilent 7890 A gas chromatogram, fitted with a G3486 A CFT flow modulator and a three-way splitter plate coupled to a flame ionisation detector and a HES 5977B quadrupole mass spectrometer with electron ionisation (Agilent Technologies Ltd, Stockport, UK). During method development, the following phases were tested as the secondary column; DB-WAX (Agilent Technologies Ltd, Stockport, UK), Rtx-200, Rtx-5MS, Rtx-1MS, BPX-50 (GC×GC Selectivity Kit, Restek Thames Ltd, Saunderton, UK) and SLB-IL111 (Sigma Aldrich, Dorset, UK). The following phases were tested as the primary column; Rtx-5MS (Restek Thames Ltd, Saunderton, UK), DB-WAX and VRX (Agilent Technologies Ltd, Stockport, UK). The column configuration for the analysis of samples was a 5% phenyl 95% dimethylpolysiloxane 30 m x 0.25 mm x 0.25 μm Rxi-5Sil MS primary column and a polyethylene glycol 4 m x 0.25 mm x 0.25 μm DB-WAX as the secondary column. Helium was used as the carrier gas and the primary and secondary column flow rates were kept constant at 0.6 and 23 mL/min, respectively. The modulation period was set to 3 s, with a fill and flush (load and inject) time of 2.799 s and 0.201 s, respectively. The restrictor from the first outlet port of the splitter plate to the FID was 1.2 m × 0.25 mm deactivated fused silica, with a constant flow of 23 mL/min and the restrictor from the splitter plate to the qMS was 0.76 m x 0.10 mm deactivated fused silica.

The FID heater was set to 250 °C, the make-up gas was purified nitrogen set to keep a constant combined restrictor and make-up flow of 25 mL/min, with a purified air flow rate of 400 mL/min and 35 mL/min of hydrogen from a Peak Scientific hydrogen generator (Trace hydrogen, Peak Scientific Instruments Ltd, Inchinnan, UK). The FID collected data at 100 Hz. The temperature of the transfer line to the qMS was kept at 250 °C and the temperature of the ion source and quadrupole were kept at 230 °C and at 150 °C, respectively. The mass scan range was *m/z* 40–300 at 10,000 u s^−1^ giving an acquisition rate of 21.5 Hz. The oven was programmed from 30 °C, held for 5 min, then heated to 80 °C at 3 °C/min, then at 5 °C/min to 250 °C and held for 10 min. Between each run a bake-out method was performed, the primary column flow was increased to 1.5 mL/min and the oven was held at 250 °C for 30 min.

The instrument was interfaced with a Markes TD-100xr thermal desorption autosampler (Markes International Ltd, Llantrisant, UK). After spiking with internal standard solution, the tubes were capped with Diff-Lok caps (Markes International Ltd) and placed in trays; typically six sample tubes per tray along with a tube loaded with the n-alkane and aromatic mixture and in every other tray another tube loaded with either the indoor air mixture or programmed test mixture. When a tube was loaded by the autosampler, it was pre-purged with carrier gas for 1 min at 50 mL/min and then desorbed at 300 °C for 5 min with a flow of 50 mL/min onto a ‘hydrophobic, general’ trap which matched the sorbent in the sample tubes (Markes International Ltd, Llantrisant, UK) held at −10 °C. The trap was then purged for 2 min at 2 mL/min before being heated at the maximum heating rate to 300 °C for 5 min with a split flow rate of 2 mL/min. Between each sample tube, a bake-out method was performed which involved an empty tube (no sorbent) being loaded by the autosampler. The empty tube was pre-purged for 1 min at 50 mL/min and desorbed at 350 °C for 10 min with a split flow rate of 50 mL/min. The trap was held a 30 °C, purged for 1 min at 50 mL/min and then desorbed at 320 °C for 5 min with a split flow rate of 50 mL/min. During each tray batch a trap blank was also analysed, the trap was purged for 2 min at 20 mL/min and then desorbed at 300 °C for 5 min with a split flow rate of 2 mL/min.

Data was acquired in MassHunter GC–MS Acquisition B.07.04.2260 (Agilent Technologies Ltd, Stockport, UK) and the data processed using GC Image™ v2.6 along with GC Project and Image Investigator (JSB Ltd, Horsham, UK).

## 3 Results and discussion

### 3.1 Method optimisation

#### 3.1.1 GC×GC parameters

Flow modulation was achieved using a microfluidic device based on Agilent’s capillary flow technology, also known as valve-based modulation or flow-diversion technology [19,20]. This modulator has been reviewed and previously used for the analysis of flavour and fragrance compounds, petrochemicals and bacterial fatty acid esters [21,22]. The device provides full transfer modulation and therefore the GC×GC method can be described as comprehensive as the entirety of the sample undergoes the two different mechanisms of separation on both columns equally while maintaining near-^1^D separation (Section 0).

The optimisation was conducted using both the indoor air mixture and breath samples. The test breath samples were collected from multiple consenting control subjects in the laboratory. Since each breath sample took approximately 10 min to acquire, the indoor air mixture allowed small modifications within the method to be tested prior to testing each major modification (e.g. column change) using a breath sample. Initial experiments consisted of analyzing both breath and the reference mixture under different modulation periods (P_M_); using different ^2^D stationary phases and ^2^D column lengths. Stationary phase selection is an important aspect of GC×GC optimisation and should be specific to the types of compounds and matrix being analysed. Breath has been reported to comprise of a complex mix of VOCs with a range of chemical moieties, e.g. branched alkanes and alkenes, aromatics, amines, aldehydes, sulphides, ketones, alcohols, acids and esters [23]. However, few published examples of a GC×GC chromatogram exist which clearly depict the separation of breath VOCs, the underlying chromatogram structure (ordering of chemically related compounds) and chemical group types present and the relative abundances. Therefore, it was necessary to test different ^2^D phases to choose an optimal column configuration. The inject (flush) time was fixed and the P_M_ varied from 1.2 to 6 s. Despite less than optimal peak shape, this gave an initial insight into the complexity of the breath matrix. From the results, approximations for the average number of peaks, extent of ‘wrap-around’, peak width and modulation ratio (M_R_) were obtained. A less conventional, reverse column configuration, with a polar wax column as the ^1^D column and a non-polar column used for the ^2^D column was also tested. However, an Rtx-5MS was favored as the primary column because of the better separation observed across the majority of compound classes (Section 3.5) and use of a non-polar primary column reduces the interaction of water with the stationary phase upon initial trap desorption, which is important for continual running of a high volume of samples. The column configuration used was a primary Rtx-5Sil MS low polarity column combined with a secondary high polarity DB-WAX column; a highly orthogonal column combination with a P_M_ of 3 s and a 4 m ^2^D column.

**Fig. 1:**
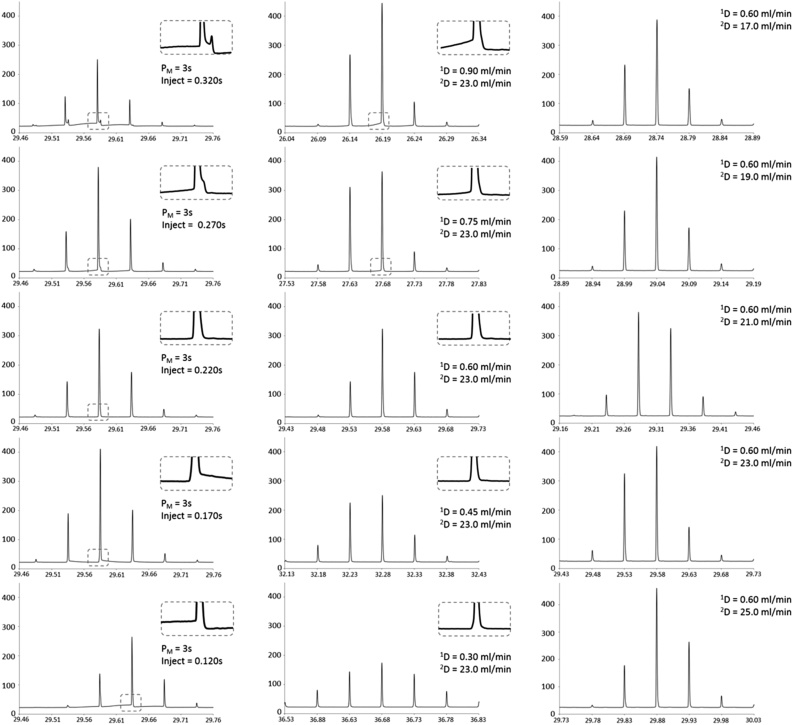
1D representation of modulated peaks of α-pinene in the reference indoor air mixture, showing the effect of modulation and flow parameters on peak shape. (Left) Effect of inject time on modulated peak shape. (Centre) Effect of ^1^D flow rate on modulated peak shape. (Right) Effect of ^2^D flow rate on modulated peak shape.

Optimisation of the modulation parameters and flow conditions were performed using the indoor air standard reference solution and laboratory breath samples. The reference mixture contained a wide range of VOCs representative of those in indoor air, hence, many of the components overlapped with those expected in breath making it a suitable reference mixture of choice for aiding method development. The mixture contained 42 compounds, which eluted across the whole chromatogram, allowing the parameters to be optimised for compounds across a range of volatilities and chemical classes. The optimisation excluded the few per-halogenated compounds present in the mixture, as these were not expected to be endogenous to breath. A summary of the compounds within the indoor air mixture and the parameters under the optimised conditions is given in Table 1. Fig. 1 shows the effects of inject time and ^1^D and ^2^D flow rate on modulated peak shape and sensitivity. The modulated peaks of α-pinene within the indoor air reference mixture were used to demonstrate the effects of each parameter in Fig. 1 because the compound eluted in the centre of the chromatogram without any co-elution in the primary dimension, allowing a clear one-dimensional depiction of the peak shapes, however, all the compounds were used to evaluate the method (Table 1). An iterative process of varying one variable at a time allowed optimal conditions to be identified within the pressure limits and requirements of the splitter plate and thermal desorption unit.

Peak shape, sensitivity and separation were highly sensitive to small changes in the load and inject intervals that make up the P_M_ as well as the primary and secondary dimension (^1^D and ^2^D) flow rates, which fill and flush the modulation channel. If the ^1^D flow rate was too fast or the load time too long, analytes eluting from the ^1^D column interfered with the previous modulation and if the ^2^D flow rate was too slow or the inject time too fast the analytes experienced inefficient re-injection; tailing peak shape and affected the separation observed in the next modulation (known as ‘wrap-around’) (Fig. 1).

The P_M_ was fixed at 3 s and the inject time varied from 0.120 to 0.320 s (in 50 ms intervals). The optimum inject time was 0.220 s, demonstrated by the shape of the modulated peaks in Fig. 1. The results showed that only a 50 ms deviation from this optimum timing was detrimental to peak shape with significant peak fronting and tailing observed 100 ms above or below 0.220 s (Fig. 1, left column).

Using a constant inject time and ^2^D flow rate, the ^1^D flow rate was varied from 0.3 to 0.9 mL/min (0.15 mL/min intervals). The optimum ^1^D flow rate was 0.6 mL/min. Lower ^1^D flow rates resulted in lower sensitivity and wider total (reconstructed) peak width; observed in Fig. 1 (centre) by an increase in the number of modulated peaks. This maximizes the potential ^2^D separation but at the detriment of the ^1^D separation, known as over-sampling [24] (Section 0). A minimum trap flow of 2 mL/min, meant higher primary column flow rates such as 0.90 and 0.75 mL/min had higher sensitivity due to a lower split upon trap desorption, but showed significant peak distortion due to breakthrough of the primary column eluent into the modulation channel. While 0.6 mL/min was below the theoretical optimum flow rate of the column, it was the highest flow rate which gave the best ^1^D separation across the chromatogram, demonstrated by the separation of toluene from toluene-d_8_ at the beginning and phenanthrene from anthracene and phenanthrene-d_10_ towards the end of the chromatogram (Fig. S2). A flow rate of 0.6 mL/min also ensured the carrier gas pressure remained within the maximum limits of the TD-100xr autosampler (60 psi) at the maximum programmed oven temperature (250 °C).

Next, the ^2^D flow rate was varied from 17 to 25 mL/min (2 mL/min intervals), keeping the inject time and ^1^D flow rate constant (Fig. 1, right). An increase in the ^2^D flow rate resulted in a small increase in peak height; a decrease in ^2^D peak width and shorter secondary dimension retention times (^2^t_R_). Narrow ^2^D peak widths are optimal as this increases overall peak capacity and a decrease in the ^2^t_R_ reduces the likelihood of wrap-around, particularly when using a longer secondary column. The ^2^D flow rate had less of an effect on performance than ^1^D flow rate and inject time, with 23 mL/min chosen as the ^2^D flow rate.

#### 3.1.2 Dual FID and qMS detection

Whilst the method optimisation was performed on both breath and the indoor air reference mixture, in the absence of a universal breath standard, the multi-component indoor air mixture was used to assess the chromatographic performance and data acquisition by dual FID and qMS detection (summarised in Table 1). Breath is highly variable, even from the same individual, making it difficult to compare metrics across samples. Reporting the metrics which demonstrate the fully optimised method using a commercially available reference mixture allows comparison of the parameters across studies.

The retention times in the primary and secondary dimensions (^1^t_R_ and ^2^t_R_) of the VOCs in the indoor air mix showed good intra-batch repeatability, with RSDs of 0.1 and 1.3% (n = 6, Table 1), removing the need to perform chromatographic alignment on samples analysed within the same tray batch [22]. Inter-batch repeatability was determined based on the retention positions on the 30 compounds present in the n-alkane and aromatic reference solution, again with low standard deviations of less than 0.01 min and 0.01 s for ^1^t_R_ and ^2^t_R_ (n = 6, average RSDs of 0.1% and 1.7%), respectively. This was reflected in the low linear retention index (LRI) variability (Fig. S3). Monitoring the variability of ^1^t_R_ and ^2^t_R_, or LRI, over time due to column degradation and its effect on separation is discussed further in section 3.2. It should be noted that ^1^t_R_ and ^2^t_R_ herein refer to the primary and secondary retention time of the modulated peak with the largest apex within the reconstructed 2D peak. Therefore, the ^1^t_R_ includes the hold time in the modulator, the ^2^t_R_ and the restrictor dead time.

Careful control of the restrictor lengths from the splitter plate using the Agilent splitter plate calculator, resulted in excellent retention position matching between the two detectors; less than 0.02 min and 0.04 s difference for ^1^t_R_ and ^2^t_R_, respectively (Fig. S4). Therefore, future investigation of the FID data for breath biomarkers (e.g. using pixel-based workflows [[25], [26], [27]]), can be easily supported by the identification of discriminatory markers using the mass spectral data based on their matching retention positions in both chromatograms. The use of dual detection also allows the use of peak templates based on mass spectral matching, a feature in GC×GC data processing software such as GC Image (JSB Ltd, Horsham, UK) and ChromSpace (SepSolve Analytical, Peterborough, UK), for the assignment of peaks across multiple chromatograms.

Valve-based modulation relies on a flow differential between the ^1^D and ^2^D columns. In order to obtain narrow peak widths and efficient transfer of the primary eluent onto the ^2^D column, the ^2^D flow rate is often very high (e.g. > 20 mL/min) creating compatibility issues with vacuum based, low flow detectors such as a mass spectrometer. Low duty cycle modulators result in reduced sensitivity, however full transfer modulators such as the ‘flow diversion’ designs outlined by Bahaghighat et al. [20] must be split to either multiple columns or detectors. In the current study, the use of a flow diversion modulator, capable of full transfer GC×GC modulation, with the ^2^D flow split between two detectors maximized the data available. A mass transfer experiment was performed to ensure full transfer during modulation using the CFT device. The n-alkane and aromatic mixture was analysed with the modulator on using the optimized parameters described herein, and then with the modulator valve off but with the remaining parameters such as column flows kept the same [28]. The sum of the modulated peak areas per compound compared with the un-modulated peak areas was within 0.25%.

The modulation ratio (M_R_) is a metric often used to assess if the peaks within a sample are efficiently modulated, or if there is under- or over-sampling. The optimal M_R_ is 2–4 with an M_R_ of at least 3 has been proposed for the analysis of trace compounds [24]. A M_R_ of < 2 results in under-sampling which results in the modulation having a detrimental effect on the ^1^D separation [20]. A M_R_ > 4 results in over-sampling which suggests the ^2^D separation has not been fully optimized [20]. The M_R_ was calculated as proposed by Khummueng, et al. [24], based on the average peak width for each compound within the indoor air mixture, given in Table 1. The average M_R_ was 3–4 (Table 1) meaning efficient modulation was achieved using the method parameters.

Analysis of the indoor air mixture showed the FID with an acquisition rate of 100 Hz recorded on average of 20–30 data points across each modulated peak (average width ˜230 ms, Table 1). Therefore, the M_R_ and acquisition rate were considered sufficient for collecting quantitative data across the broad dynamic range of VOCs present in breath (Table 1) [29]. The FID data was supported by a fast-scanning qMS with a scan rate of 21.5 Hz. Table 1 shows on average 6–8 full mass spectral scans per modulated peak width which was sufficient for mass spectral identification of breath VOCs [29].

### 3.2 Retention behavior and column degradation

**Fig. 2:**
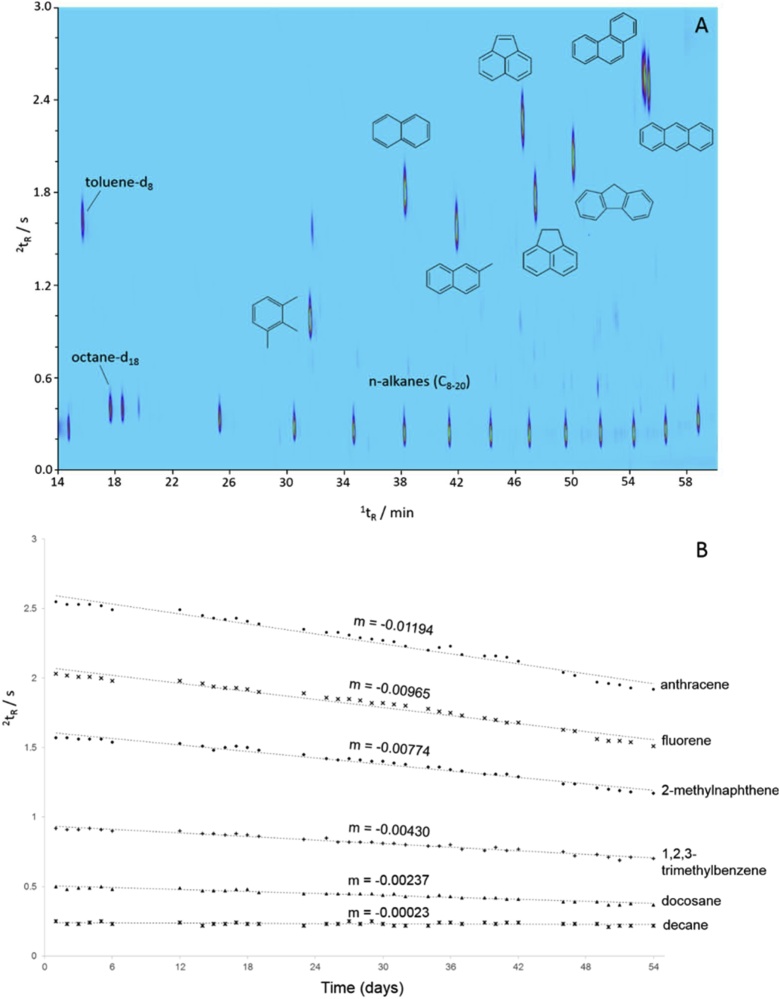
(A) Flow diagram of the detailed sample history, processing and data being recorded at every stage of breath collection and analysis to ensure high quality breath analysis. 1) Tube weights and number of heat cycles recorded to monitor sorbent integrity; 2) tube conditioning and batch blanks; 3) kit preparation; 4) ReCIVA breath collection using SOPs following comprehensive training, producing ReCIVA metadata; 5) dry purging and IS spiking; 6) daily instrument checks and analysis of samples and reference mixtures. (B) ReCIVA quality control data showing gated breath collection, controlling the breath fraction, flow rate and total volume. (C) Three chromatograms collected per sample including breath, room air and air supply by TD-GC×GC-FID/qMS.

For on-going analyses within a large-scale study, it was important to monitor the quality of separation and the gradual change of retention positions, which occur over time with column degradation. Separation performance was monitored by the routine analysis of an n-alkane and aromatic mixture at the beginning of each tray batch (Fig. 2A). The retention positions of the n-alkanes allowed monitoring of the ^1^D separation, while the separation between the n-alkanes and aromatics with increasing ring number helped monitor the ^2^D separation. Fig. 2B shows the change in ^2^t_R_ (Δ^2^t_R_) as a function of time, proportional to the number of completed runs. The Δ^2^t_R_ over a period of 54 days, represented by the regression coefficient *m*, gradually increased with respect to initial ^2^t_R_, (*m* = -0.00023 for decane compared with *m* = -0.01194 for anthracene). This was expected within an orthogonal system, with ^2^D column degradation having the greatest effect on the classes of compounds which experience greatest affinity with the wax column (Fig. 2). The data were used to inform the operator when to replace the ^2^D column and can be used to inform chromatographic alignment during data processing.

Chromatographic alignment is a crucial step within a metabolomics data workflow, especially for second-order data, such as the GC×GC-FID (or GC-IMS) data reliant on unique retention positions. Alignment can be achieved based on the traditional approach of calculating retention indices for each feature based on the positions of the n-alkane standards (Fig. S3), or based on an image analysis approach using the n-alkane and aromatic standards as reference points for transformation by image registration.

### 3.3 Breath collection and analysis

**Fig. 3:**
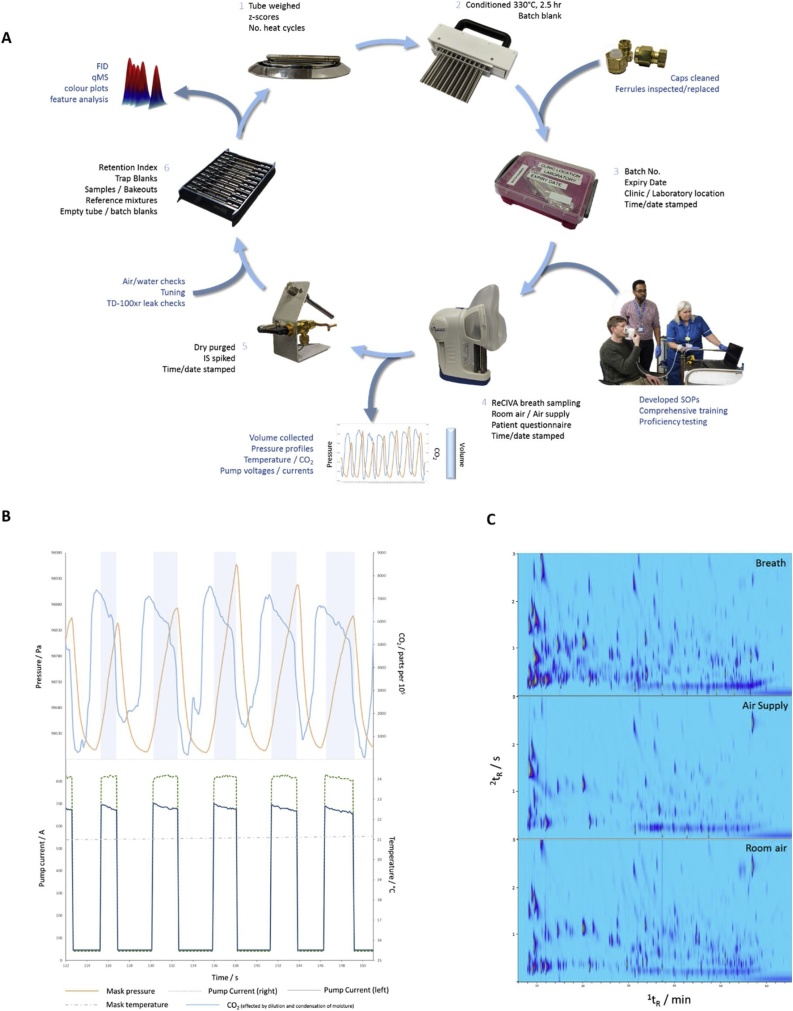
(A) GC×GC chromatogram of n-alkane and aromatic reference mixture monitoring retention behavior across available chromatographic space. (B) Change in secondary retention time (*m*) over time with column degradation and runs completed.\.

A high quality breath sample is defined by a good record of sample history as much as good sampling technique. Good sampling technique requires comprehensive training and SOPs for clinical research staff (Fig. 3A) and when using sorbent tubes includes, but not limited to, ensuring a secure seal when capping the tubes; always handling the tubes with gloves; not handling the ends of the tubes; not leaving the tubes uncapped for any length of time except during sampling; once sampled keeping the tubes refrigerated in a low-odor environment; conditioning face masks or silicon-based products before use; taking regular environmental samples e.g. sampling the room air and air supply.

Breath is highly influenced by environment, diet and fitness. Best analytical practice, under laboratory conditions, would attempt to control as many variables as possible e.g. controlled diet. However, for breath analysis to be readily adopted within large-scale clinical studies a balance must be maintained between best analytical practice and what is practical and feasible within a clinical environment and patient treatment pathway. Otherwise the time-cost associated with controlling every influencing factor would be greater than the non-invasive advantages of breath analysis and make the technique clinically unfeasible. A high quality breath sample can still be obtained but it must be accompanied by a detailed sample history, where the data can be integrated as part of the analysis to account for confounding factors. Therefore, within the current method, a detailed sample history was recorded at every stage of the sample collection and analysis (Fig. 3).

For example, from the beginning of the preparation process, the number of heat cycles and weight for each tube was recorded prior to conditioning and an individual z-score calculated for each tube. In combination with the flow rate data measured during dry purging and spiking, this provided a measure of sorbent tube integrity. A significant loss in mass indicated if the tube had been damaged during transit or in-clinic, resulting in loss of sorbent and a significant increase in mass indicated inefficient tube desorption or contamination.

Each batch of tubes conditioned at 330 °C for 2.5 h, were given a batch number and a batch blank taken, stored and prepared in the same way as the breath, room air and air supply samples. This monitored for contamination from the beginning of the preparation process for each batch of tubes. All conditioned tubes were given an expiry of 2 weeks, this ensured a controlled, continuous flow of tubes to and from the hospital with regular monitoring and conditioning. Before being packed into kits, each box, tubing for the air supply and set of caps were washed and oven dried. Face masks (Owlstone Medical Ltd) were conditioned at 180 °C overnight before being delivered to clinic, this reduced the background level of siloxanes. The face masks were given an expiry of 2 weeks after which unused masks were then reconditioned.

A date and time stamp was recorded at conditioning, sampling, dry purging and analysis. This ensured any variation caused during sample handling owing to storage time could be accounted for. During a sample collection, the time; operator; study number; tube number; air supply flow rate; location and volume collected was recorded on an electronic transfer sheet alongside the ReCIVA metadata file. The transfer sheet was barcoded and a copy automatically emailed to the laboratory on completion of the sample collection. In addition to patient history and clinical data collected as part of the study, a patient questionnaire on recent diet, medication, lifestyle and hygiene was collected. The transfer sheet and patient questionnaire ensured potential confounding factors such as last meal, circadian rhythm, oral hygiene and smoking could be accounted for in the discovery workflow.

### 3.4 Sample viability

The criteria for a high quality breath VOC measurement is difficult to refine to a single metric or index, it requires routine quality control in several areas to verify the correct collection (e.g. volume, fraction, technique, storage) and analysis (e.g. peak shape, resolution, response) of samples; standard operating procedures (SOPs) to ensure consistent results with regular training and proficiency testing; and a detailed sample history. Therefore, a subset of samples were assessed to determine if the relative levels of abundant, endogenous compounds present in breath (relative to the environmental levels) could be used as a quick screening method to ensure a sampling tube contained a viable breath sample. Checking sample viability allowed quick visualisation of potential errors, such as operator error, incorrect tube labeling, ReCIVA functionality errors, faulty air supply, or tube desorption error. Tubes with questionable viability were then investigated further against the other quality measures such as the storage time, the metadata recorded by the ReCIVA device and the other metrics discussed herein, helping to screen the data before being used within a discovery analysis workflow.

This approach was adapted from a routine check, which was part of the protocol for analysing breath by PTR-TOF-MS, which provides real-time breath data in less than 1 min. To ensure the breath sample had been recorded correctly (e.g. sample lines connected and no blockages in the line or in-line filter) without increasing the time required to acquire a sample, the ions corresponding to protonated acetone and isoprene were monitored.

**Fig. 4:**
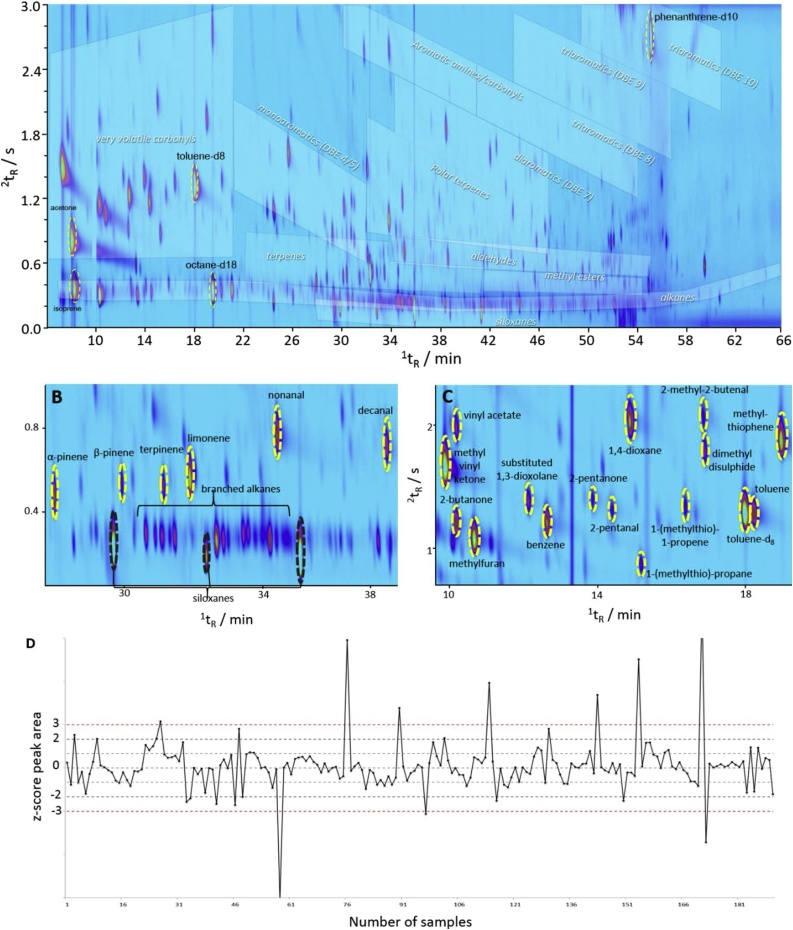
(A) An example chromatogram (portrayed as colour plot) of an exhaled breath sample captured using the ReCIVA and analysed by thermal desorption GC×GC, showing the separation of volatile organic compounds present in breath and regions of different chemical classes. (B and C) Example regions of a chromatogram showing the separation of volatile n-alkanes, carbonyls, siloxanes, sulphur-containing compounds and terpenoids. (D) Quality control chart of the internal standard toluene-d_8_ for monitoring system performance over time.

The acetone and isoprene peaks were integrated across a subset of 460 samples, including breath, air supply samples and room air samples. Briefly, the chromatograms were aligned in batches using the retention position of the 30 components within the n-alkane and aromatic solution, run at the beginning of each tray, as reference points and the images registered using a 2^nd^ order polynomial transformation. A smart template was created including the aligned retention positions of acetone and isoprene, allowing integration of the two VOCs across all 460 chromatograms. Quality control charts used monitor the peak area of the internal standards, toluene-d_8_ and octane-d_18_ showed good reproducibility (Figs. 4D and S5).

As expected, for a viable breath sample, the relative abundance of acetone and isoprene were greater in the exhaled breath samples than the room air and air supply samples (Fig. S6) with the ratio between the two VOCs being significantly greater in breath samples. Quality control charts of the acetone and isoprene peak areas in the air supply and breath samples, allowed the identification of problematic samples (Fig. S7A and B). For example, cross-comparison of this data with the metadata stored by the ReCIVA breath sampler and transfer sheet notes, showed for three of the breath samples that lay outside 3σ (z >3), only 9–26 mL out of the 1 L collection volume had been collected, with an error in the pump current that was responsible for drawing the breath through the sorbent tubes. The agreement between the VOC data and the other quality control data highlights the level of acuity that can be derived from the breath data collected within the study. It also highlights the method developed herein including sample collection and analysis had a success rate of >97%. The movement towards standardisation within the breath research community could benefit from the use of indexes or quality control records in the collection and analysis of breath samples.

### 3.5 Analysis of exhaled breath VOCs

Despite an increasing number of studies that have explored the potential of GC×GC for the analysis of exhaled breath, previous reports lack details of method development and the demonstration of a robust methodology for the analysis of breath integrated as part of a large-scale clinical study. Few studies provide necessary details for replication and this limits the adoption of the technique within the breath research community.

The main, and often only, metric used to assess the sampling and chromatographic performance in previous studies, utilizing GC×GC for the analysis of exhaled breath biomarkers, has been the total number of VOCs detected. This value has been represented as the number of different VOCs detected either across all the samples; in a percentage of samples (e.g. 50 or 100% of patients); or the highest number of VOCs detected in a single sample. For example, Phillips et al. [13] detected over 2000 different VOCs across 34 subjects using GC×GC, with 1000 VOCs detected in 50% of subjects and 95 VOCs in 90% of samples [15]. Das et al. [14] reported that 1379 VOCs were detected in a single breath sample analysed by GC×GC, from a group of 47 subjects, with 129 and 41 VOCs detected in 50% and 100% of subjects, respectively [16]. These numbers are impressive but do not necessarily demonstrate the enhanced separation afforded by GC×GC or infer the quality of the sample collected. Whilst the highest number of VOCs in a single breath sample analysed by GC×GC was substantially higher than the typical number reported by GC–MS, between 150–250 VOCs [23], results of peak detection and deconvolution software applied to breath data acquired by GC—MS have reported similar total numbers of VOCs across samples, with up to 3481 VOCs previously detected by GC–MS [23].

It should be noted, that the detection of a VOC often constitutes the detection of a chromatographic feature above the thresholds and parameters set within the automated peak detection and/or deconvolution algorithm in the software used to process the data, with a chemical name assigned based on a similarity threshold with a mass spectral library match. This allows recursive feature analysis of the hundreds of peaks, extracting each unique feature across all samples to input into a multivariate analysis. However, reference compounds or more rigorous interrogation of the mass spectra and retention position should be used to confirm the identity of the small number of peaks which have later been shown to be significant biomarker candidates following statistical processing.

For comparative purposes, in the current study, a similar automated peak detection tool was used to process a subset of FID data. The number of VOCs across 117 different, quality controlled, breath samples varied on average from 250 to 600, similar to previous GC×GC studies [30]. Since these numbers were greater than the average number of VOCs typically reported for GC—MS without the added advantages of mass spectral deconvolution, this metric highlights the chromatographic advantage of the current GC×GC method over GC—MS. Automated methods for the deconvolution of complex mixtures analysed by GC—MS and GC×GC—MS often requires a skilled operator to manually review the results; with the total number of peaks detected being dependent on the parameters and thresholds used. Yet the number of VOCs detected in the FID data alone, herein, was based solely on one signal-to-noise threshold, with the high number of peaks observed reflecting the optimised chromatography (Sections 3.1 and 3.2). GC×GC data also has the added advantage of being able to immediate observe the group separation of different chemical classes present in the sample, from visual inspection of the two-dimensional image (Figs. 4 and S8).

However, it is suspected that the biological variation between patients; the variation caused by lifestyle (e.g. smoking, hygiene and eating); the sampling procedure and sorbent tubes used and the variation caused by the levels of environmental VOCs at the time of sampling, have a significant effect on the total number of VOCs reported, perhaps more so than the separation capacity of the instrumentation used. Ultimately, the advantages of one technique compared with another will become more evident in the discovery and quality of biomarker candidates identified, as well as the ease of targeted analysis following the discovery of a set of compounds of interest. The results of the discovery analysis of breath biomarkers in acute cardiorespiratory disease, including method validation and the data workflow for handling the large number of chromatographic data files will follow on completion of the discovery dataset. However, the rigor and metrics used to assess the GC×GC performance and sample collection herein ensures quality in the data collected, and will be used for the standardisation of different breath analysis techniques.

Fig. 4 shows the clear separation of many VOCs present in exhaled breath. The chemical ordering of analytes in the chromatogram showed non-polar constituents e.g. hydrocarbons, eluting in the lower region of the chromatogram. The separation of different chemical classes and assignment of VOCs was supported by the analysis of >100 reference VOCs (Fig. S5). The non-polar compounds present in breath samples included an abundance of branched alkanes (e.g. 2-methylbutane), with unsaturated alkenes (e.g. isoprene) and cycloalkanes (e.g. methylcyclohexane) eluting just above (Fig. 4A and B). The separation of the non-polar analytes from the rest of the matrix is important because volatile n-alkanes and methyl- branched alkanes have been reported as tentative markers of oxidative stress [31,32]. Reactive oxygen species (e.g. hydrogen peroxide) produced during energy metabolism within mitochondria are proposed to react with polyunsaturated fatty acids and produce methyl- branched alkanes as a result of lipid peroxidation, which are then excreted in breath [33]. Whilst separation of the individual branched hydrocarbon isomers is achievable (Fig. 4B), structural assignment of individual isomers remains an analytical challenge owing to the numerous possible configurations and similarity of the EI mass spectra. However, given their proposed origin, its unlikely individual isomers will be significant markers on their own. The group-type separation afforded by GC×GC of the chemical class as a whole could be a useful proxy for monitoring oxidative stress, previously proposed to be a marker of age [32], heart transplant rejection [31] and breast cancer [34].The lower region of the chromatogram contained the siloxanes from both the disposable face mask and from the analytical column and inlet transfer line (Fig. 4B). Column bleed was observed towards the end of the chromatogram during the isothermal hold at the maximum temperature. The siloxanes from the environmental air and face mask appeared as abundant peaks at regular intervals in every breath sample, the peaks had lower ^2^D retention positions and characteristic mass spectra making them easy to distinguish from endogenous breath compounds.

The middle region of the chromatogram was dominated by saturated, unsaturated and cyclic carbonyls such as aldehydes (e.g. hexanal, nonanal), ketones (e.g. acetone, methyl vinyl ketone, butan-2-one), furans (e.g. furan-3-methanol), esters (e.g. ethyl acetate) and sulphides (e.g. carbon sulphide, dimethyl disulphide) (Fig. 4C). Aldehydes such as hexanal and nonanal have been reported as tentative markers of COPD [35] as well as lung cancer [36], again present as secondary products of oxidative stress via lipid peroxidation of fatty acids in epithelial pulmonary cells, such as linoleic acid and arachidonic acid [37]. Sulphur-containing compounds such as sulphides have been associated with liver function impairment, produced as part of the degradation of methionine [38]. Assignment of chemical identities discussed herein was made based on the analysis of reference compounds, summarized in the map of standards (Fig. S8); elution order; and mass spectral library matching and interpretation.

The top region of the chromatogram had a lower peak density with only the most polar analytes eluting late in the second dimension, such as aromatic carbonyls (e.g. benzaldehyde, acetophenone), amines, alcohols (e.g. isopropanol, trimethyl silanol) and phthalates (Figs. 4 and S8). Compounds such as benzaldehyde and isopropanol have been proposed as both tentative markers for disease but also identified as common constituents of room or environmental air samples. For example, isopropanol is present in many disinfecting products and therefore may be present in high concentrations within clinical environments [39].

A P_M_ of 3 s gave minimal wrap-around in the majority of breath samples despite the relatively long secondary column length. However, owing to the high variability of breath and its susceptibility to environmental influence, occasionally a particularly polar compound, most likely exogenous to the underlying metabolism was present and caused slight wrap-around.

### 3.6 Instrumental setup

When assessing the reproducibility of a method and the ease of adoption for future applications, such as the use of GC×GC for biomarker discovery in large clinical studies and trials, it is germane to assess not only the quality of the data but also to consider the instrumental setup and the trade-off between analytical, practical and economic requirements. The method development was driven by the needs of the clinical application and as such it was a constrained optimisation (Table 2), unlike studies that focus solely on chromatographic theory and optimal performance.

**Table 2:** Benefits and constraints of TD-GC×GC-FID/qMS analytical instrumentation.

Instrumentation	Benefits	Constraints	Considerations
Sorbent tubes and thermal desorption autosampler	High analytical capacity Low storage requirements High storage stability under adequate conditions (brass caps, dry purged, cooled) Low background artefacts	Maximum carrier pressure 60 psi Minimum trap flow 2 mL/min Retention of water on sorbent Sorbent selectivity and volatility range	Max pressure limits ^1^D and ^2^D flow rate combinations Reduced sensitivity due to necessary low ^1^D rates creating increased split Incorporate dry purge step
Flow modulator	Inexpensive, low consumables cost Easy to use, few connections Suitable for volatile analysis	Requires high ^2^D flow rate Broader modulated peak widths	Compatibility of detector(s) with high column flow rates Acquisition rate of detector(s) to capture modulated peaks
Fast scanning qMS	Cost effective Fast scanning (20,000 amu/s) Suitable for targeted analysis after biomarker identification	Compatible with < 1.5 mL/min flow rates Max acquisition rate <40 Hz Mass spectral skewing	Need to split ^2^D flow to avoid over-pressurising detector Setup to either accommodate quantitative or qualitative analysis Sensitivity changes with acquisition rate and split column flow
FID	Cost effective Compatible with flow modulator High acquisition rate Broad dynamic range	No mass spectral assignment	Setup to either accommodate quantitative or qualitative analysis Need supporting mass spectrometer for identification of unknowns
Splitter plate	Enables multiple detectors Easy to use Offers control of split ratio between detectors	Provides constant flow to one detector Requires a constant positive pressure from auxiliary EPC	Change in sensitivity due to split Sensitivity of the secondary detector(s) without constant flow control Alignment of results requires balanced restrictor lengths

The optimal sampling method for a large-scale study allows for the pre-concentration of trace VOCs (*analytical*), reliable storage of samples (*analytical/practical*) and is readily automated for higher throughput (*practical/economic*). Therefore, the chosen method for offline sample collection herein were sorbent tubes. The ReCIVA breath sampler allowed the direct trapping of breath VOCs onto sorbent tubes which were compact with low storage requirements; could be dry purged to reduced water for improved storage stability over a period of a few weeks [40]; and could be used in conjunction with a thermal desorption autosampler unit which provided the analytical capacity required for large recruitment targets within clinical studies. The limitations of polymer bags are well documented; aside from the practical implications of storing large inflated bags, the bags contribute high background levels of acetamide and other artefacts as well as permit diffusion of analytes of interest out of the bag and contaminants into the bag [41,42]. However, polymer bags are much cheaper and don’t require specialist sampling devices such as the ReCIVA device.

Flow modulation is suited for the analysis of highly volatile analytes, such as those in breath, because the modulation is independent of temperature. Consequently, the technology is more affordable than cryogenic devices, comprising only of a capillary channel and microfluidic switch. This has resulted in an increase in the use of flow controlled devices, especially within metabolomics studies e.g. in investigating the ‘volatolome’ from urine [43,44] and serum [3,45] as well as flavour and fragrance applications [28,46]. Whilst thermal modulation still has its advantages, producing narrower peak widths during modulation and allows more optimal column flows, the suitability of flow modulation for the analysis of VOCs, its ease of use (during routine daily analysis) and affordability made it a good choice of modulation for the current breath analysis method.

## 4 Conclusions

This study reports a new method, developed for the analysis of exhaled breath VOCs by thermal desorption coupled to a flow modulated GC×GC with dual FID and MS detection. To the best of the authors’ knowledge, this is the first application of flow modulation GC×GC, along with dual detection (GC×GC-qMS/FID), for the analysis of exhaled breath VOCs, for integration as part of a biomarker discovery phase within a large-scale clinical study. The use of flow modulation, fast scanning quadrupole mass spectrometer, supported by flame ionisation detection, and automated thermal desorption unit allowed the continuous analysis of breath and environmental samples to meet the high clinical recruitment targets. The sample collection protocol was shown to be clinically viable and enabled bedside sampling and the use of sorbent tubes integrated well into a robust sample pathway; maintaining low storage costs, high sample stability and compatibility with automated analysis. Evaluation of the analytical setup and assessment of the newly optimised method demonstrates the novel potential of GC×GC for the analysis of breath VOCs, advancing the movement towards standardisation within the field and highlights its applicability for large-scale studies in breath metabolomics.
