# Supplementary material for: Breath analysis by two-dimensional gas chromatography with dual flame ionisation and mass spectrometric detection – Method optimisation and integration within a large-scale clinical study
Source: J Chromatogr A. 2019 Jun 7;1594:160–72. doi: 10.1016/j.chroma.2019.02.001 (PMC6491496; doi:10.1016/j.chroma.2019.02.001)
Supplement: Supplementary file 1 [file mmc1.docx]

Supplementary Material

Breath analysis by two-dimensional gas chromatography (GC×GC) with dual flame ionisation and mass spectrometric detection – method optimisation and integration within a large-scale clinical study

Michael J. Wilde^1^, Rebecca L. Cordell^1^, Dahlia Salman^2^, Bo Zhao^3^, Wadah Ibrahim^3,4^, Luke Bryant^1^, Dorota Ruszkiewicz^2^, Amisha Singapuri^3,4^, Rob Free^3^, Erol A. Gaillard^5^, Caroline Beardsmore^5^, C. L. Paul Thomas^2^, Chris E. Brightling^3,4^, Salman Siddiqui^3,4^, Paul S. Monks^1^

^1^ Department of Chemistry, University of Leicester, University Road, Leicester, LE1 7RH, UK

^2^ Centre of Analytical Science, Loughborough University, Epinal Way, Loughborough, LE11 3TU, UK

^3^ Leicester NIHR Biomedical Research Centre (Respiratory theme), Glenfield Hospital, Groby Road, Leicester LE3 9QP

^4^ College of Life Sciences, Department of Infection, Immunity and Inflammation, University of Leicester, University Road, Leicester, LE1 7RH, UK

^5^ Paediatric Clinical Investigation Centre, Leicester NIHR Biomedical Research Centre (Respiratory theme), University of Leicester, Leicester Royal Infirmary, Leicester, LE2 7LX, UK


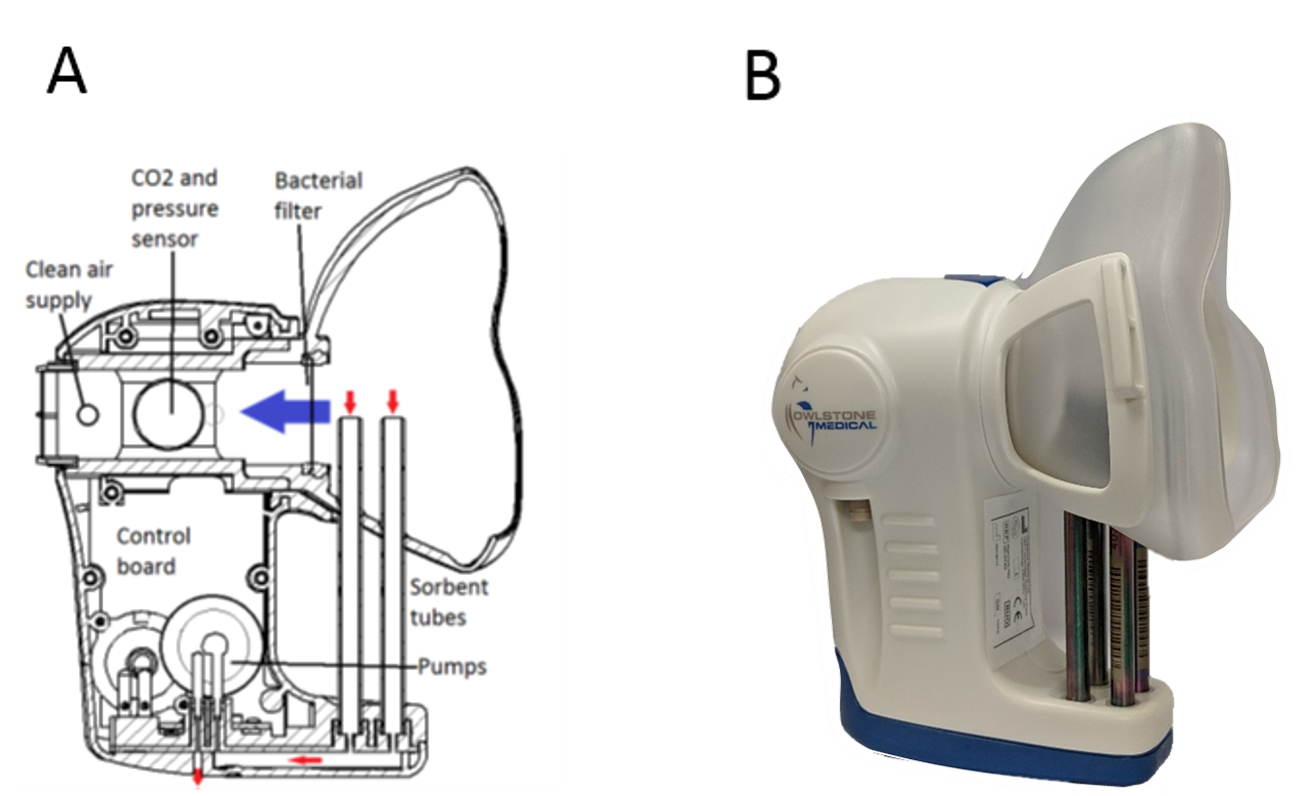


Figure S1: (A) Schematic and (B) photograph of ReCIVA breath sampler comprised of face mask, sorbent tubes, pumps and pressure sensors alongside breath profile data used for gated collection of breath VOCs [1] (see Figure 3B).


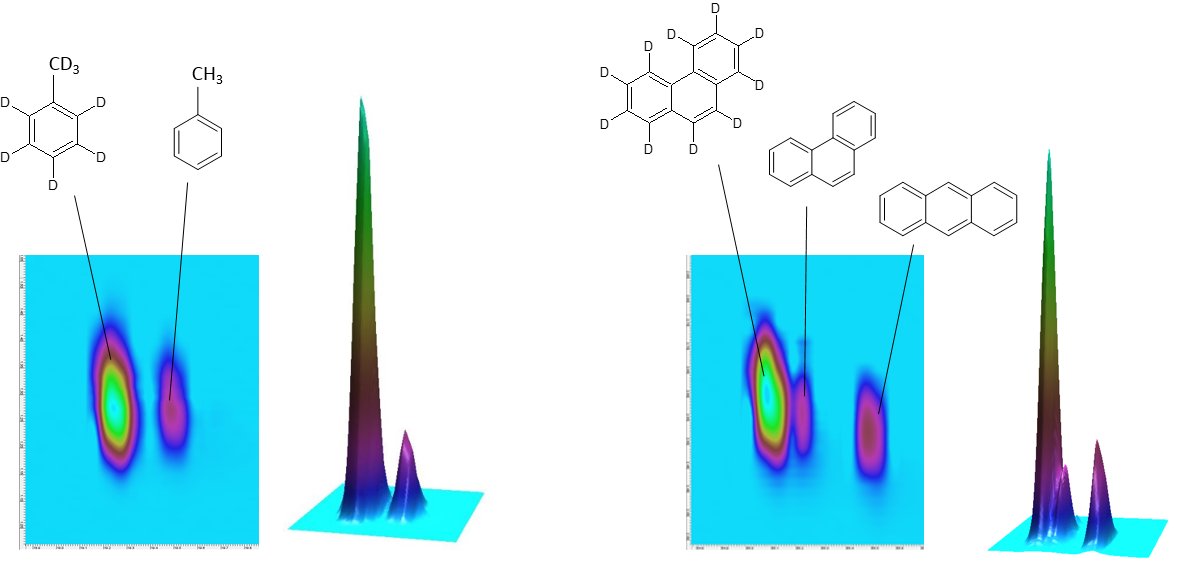


Figure S2: Separation across the chromatogram monitored using deuterated pairs; (A) toluene and toluene-d_8_ and (B) phenanthrene and phenanthrene-d_10_.


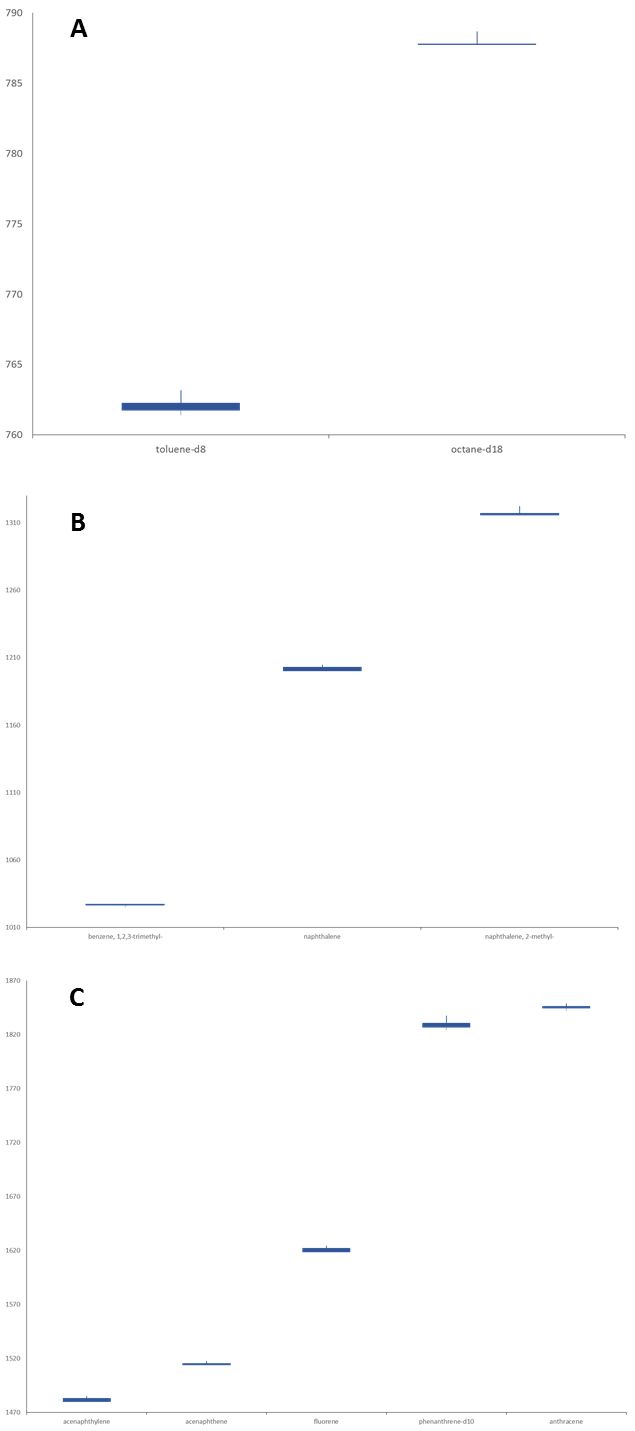


Figure S3: Linear retention index variability for aromatic compounds (n = 58, RSD <0.2%).


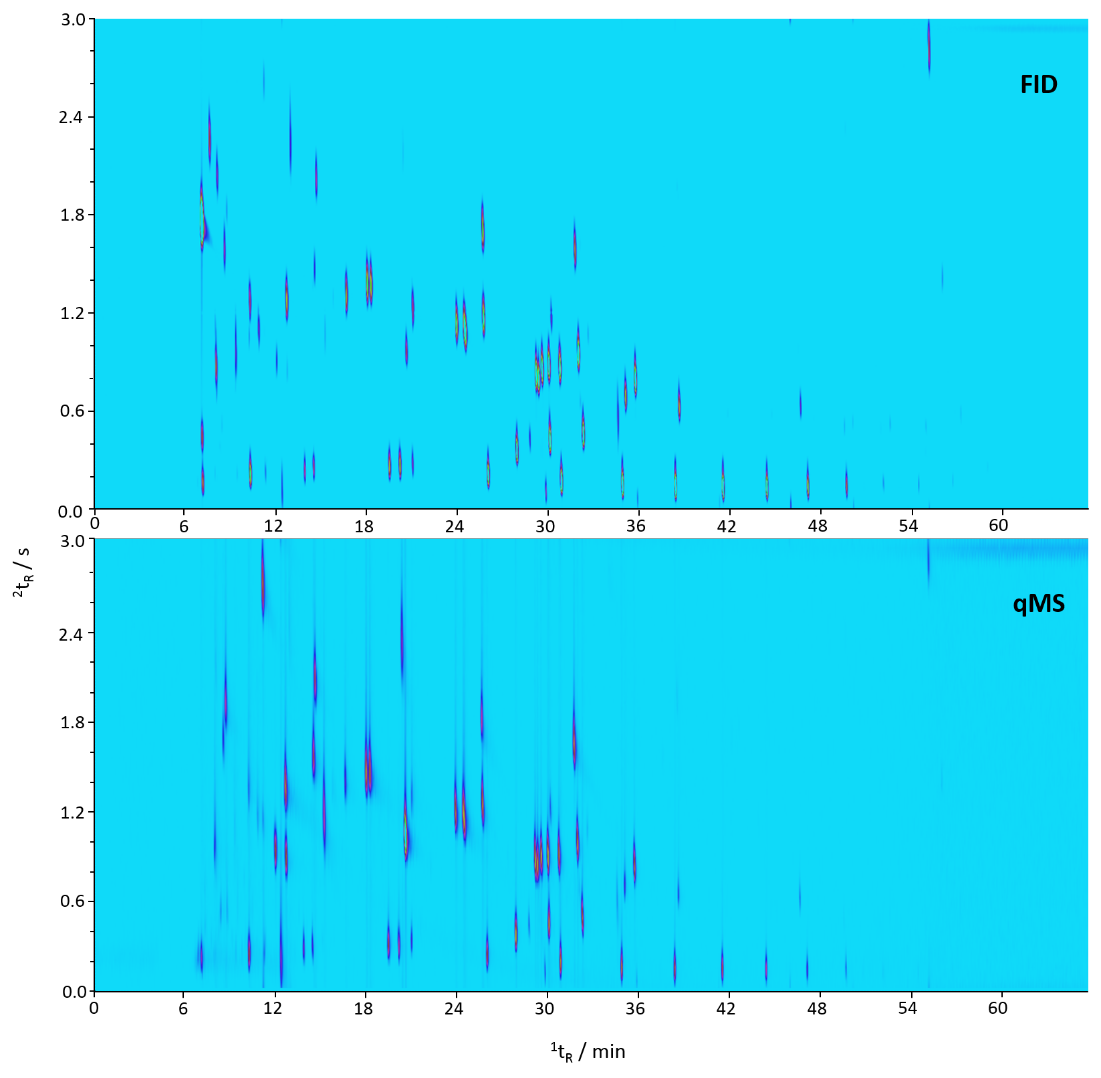


Figure S4: GC×GC-FID/qMS chromatograms of indoor air multi-component mixture.


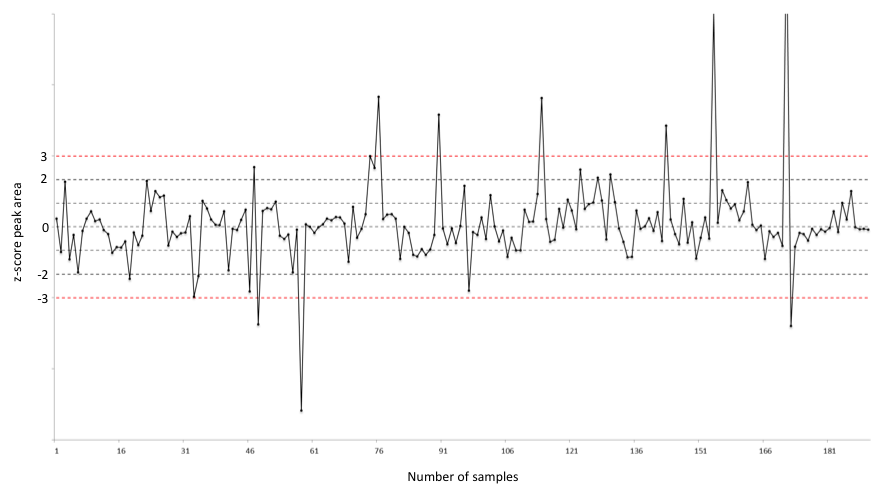


Figure S5: Quality control chart showing the z-score normalised peak area of the internal standard octane-d18 in breath samples.


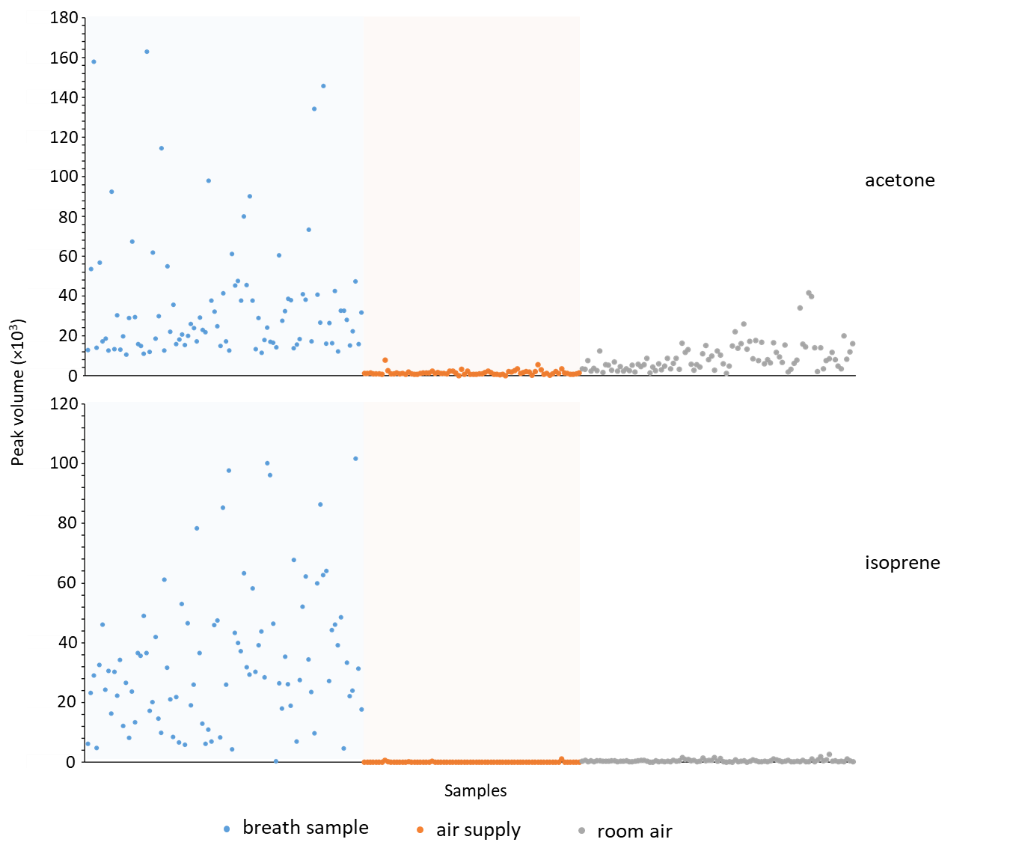


Figure S6: FID peak volumes for (top) acetone and (bottom) isoprene in breath, air supply and room air samples.


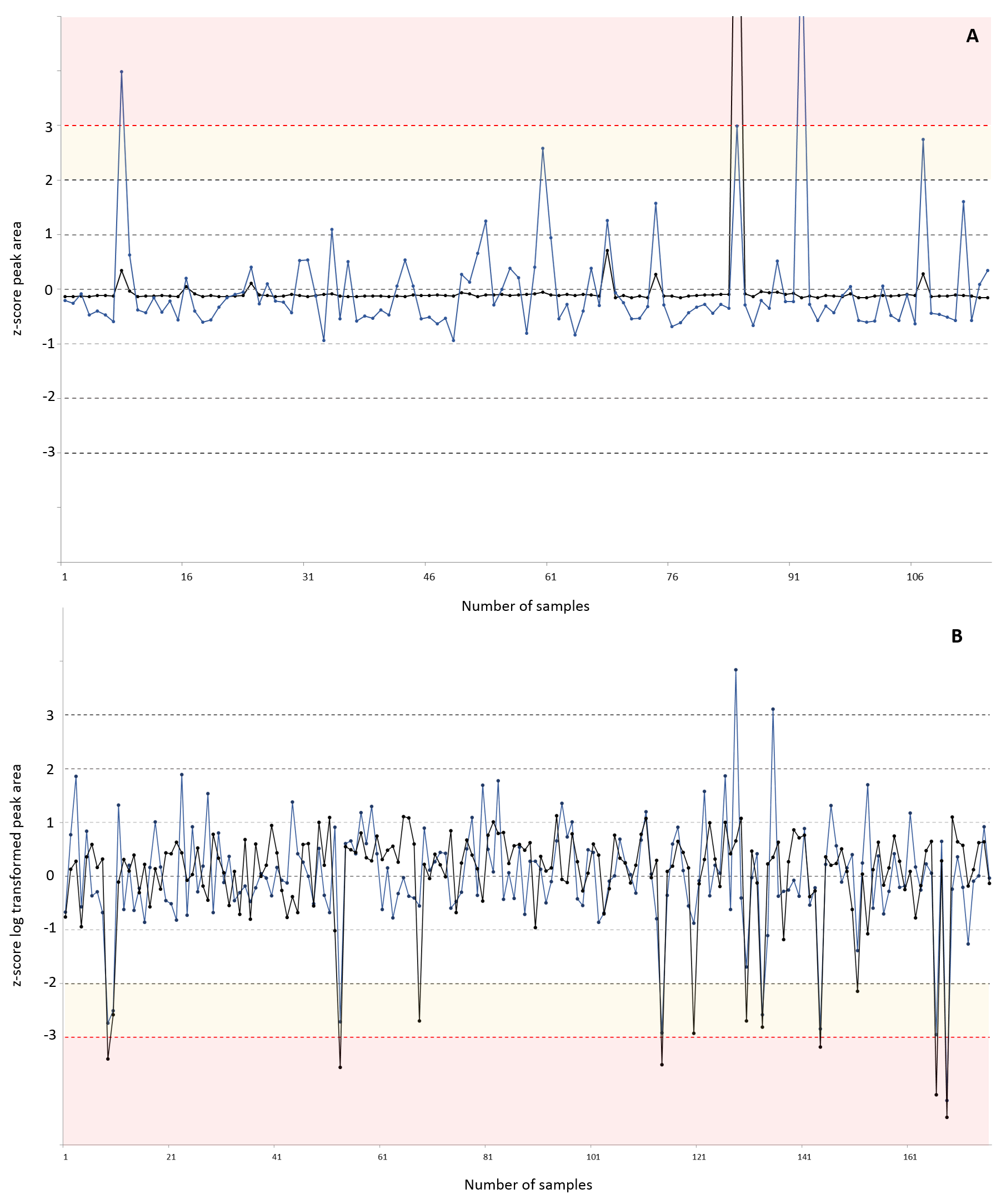


Figure S7: Quality control charts displaying the z-score normalised peak area of acetone and isoprene in (A) the air supply and (B) breath samples.


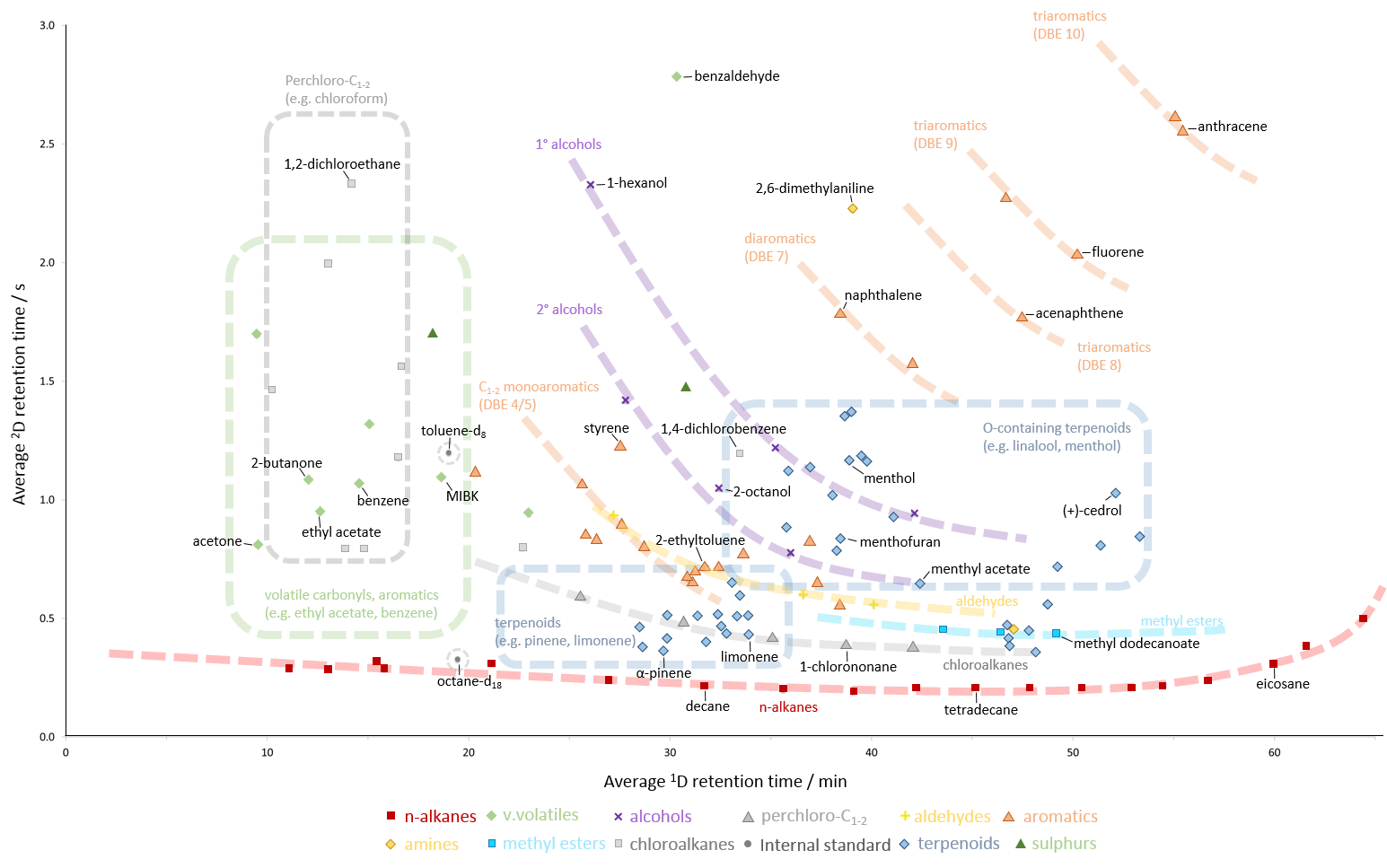


Figure S8: Graphical representation of a chromatographic map of the reference compound mixtures, showing the separation of over 100 VOCs and the different regions of chemical classes.

Table S1: Summary of compounds in the n-alkane and aromatic reference mixture.

| Compound |  |  | Concentration (µg/mL) |
| --- | --- | --- | --- |
| n-hexane |  |  | 20 |
| n-heptane |  |  | 20 |
| n-octane |  |  | 20 |
| n-nonane |  |  | 20 |
| n-decane |  |  | 20 |
| n-undecane |  |  | 20 |
| n-dodecane |  |  | 20 |
| n-tridecane |  |  | 20 |
| n-tetradecane |  |  | 20 |
| n-pentadecane |  |  | 20 |
| n-hexadecane |  |  | 20 |
| n-heptadecane |  |  | 20 |
| n-octadecane |  |  | 20 |
| n-nonadecane |  |  | 20 |
| n-eicosane |  |  | 20 |
| n- heneicosane |  |  | 20 |
| n-docosane |  |  | 20 |
| 1,2,3-trimethylbenzene |  |  | 10 |
| naphthalene |  |  | 10 |
| 2-methylnaphthalene |  |  | 10 |
| acenaphthylene |  |  | 10 |
| acenaphthene |  |  | 10 |
| fluorene |  |  | 10 |
| phenanthrene |  |  | 10 |
| anthracene |  |  | 10 |

Table S2: Summary of compounds in the GC programmed test mixture.

| Compound |  |  | Concentration (µg/mL) |
| --- | --- | --- | --- |
| 2,3-butanediol |  |  | 13.5 |
| decane |  |  | 7.1 |
| dicyclohexylamine |  |  | 8.0 |
| 2,6-dimethylaniline |  |  | 9.7 |
| 2,6-dimethylphenol |  |  | 8.2 |
| 2-ethylhexanoic acid |  |  | 10.0 |
| methyl decanoate |  |  | 10.8 |
| methyl laurate |  |  | 10.6 |
| methyl undecanoate |  |  | 10.8 |
| nonanal |  |  | 7.0 |
| 1-octanol |  |  | 9.3 |
| undecane |  |  | 7.3 |

Table S3: Summary of additional reference compounds in chromatographic map of standards (Figure S5) for chemical assignment and evaluating class separation

| Compound |  | Concentration (µg/mL) |  | ^1^t_R_ / min |  | ^2^t_R_ / s |
| --- | --- | --- | --- | --- | --- | --- |
| 1-chlorohexane |  | 61.5 |  | 25.5 |  | 0.6 |
| 1-hexanol |  | 57.0 |  | 26.1 |  | 2.3 |
| 2-heptanol |  | 57.2 |  | 27.8 |  | 1.4 |
| sabinene |  | 10.0 |  | 28.5 |  | 0.5 |
| sabinene hydrate |  | 10.0 |  | 28.6 |  | 0.4 |
| α-pinene |  | 10.0 |  | 29.7 |  | 0.4 |
| terpinolene |  | 10.0 |  | 29.8 |  | 0.5 |
| camphene |  | 10.0 |  | 29.9 |  | 0.4 |
| 1-chloroheptane |  | 61.7 |  | 30.7 |  | 0.5 |
| β-myrcene |  | 10.0 |  | 31.4 |  | 0.5 |
| β-pinene |  | 10.0 |  | 31.8 |  | 0.4 |
| α-phellandrene |  | 10.0 |  | 32.3 |  | 0.5 |
| 2-octanol |  | 57.4 |  | 32.5 |  | 1.1 |
| 3-carene |  | 10.0 |  | 32.5 |  | 0.5 |
| α-terpinene |  | 10.0 |  | 32.8 |  | 0.4 |
| p-mentha-1,3,8-triene |  | 10.0 |  | 33.1 |  | 0.7 |
| eucalyptol |  | 10.0 |  | 33.4 |  | 0.6 |
| ocimene |  | 10.0 |  | 33.8 |  | 0.5 |
| (R)-(+)-limonene |  | 10.0 |  | 33.9 |  | 0.4 |
| 1-chlorooctane |  | 61.3 |  | 35.1 |  | 0.4 |
| 1-octanol |  | 58.1 |  | 35.3 |  | 1.2 |
| (+)-fenchone |  | 10.0 |  | 35.8 |  | 0.9 |
| linalool |  | 10.0 |  | 35.9 |  | 1.1 |
| 5-nonanol |  | 57.5 |  | 36.0 |  | 0.8 |
| fenchol |  | 10.0 |  | 37.0 |  | 1.1 |
| camphor |  | 10.0 |  | 38.0 |  | 1.0 |
| menthone |  | 10.0 |  | 38.2 |  | 0.8 |
| menthofuran |  | 10.0 |  | 38.5 |  | 0.8 |
| isoborneol |  | 10.0 |  | 38.6 |  | 1.4 |
| 1-chlorononane |  | 60.9 |  | 38.8 |  | 0.4 |
| (-)-borneal |  | 10.0 |  | 38.9 |  | 1.4 |
| menthol |  | 10.0 |  | 38.9 |  | 1.2 |
| α-terpineol |  | 10.0 |  | 39.6 |  | 1.2 |
| isoterpinolene |  | 10.0 |  | 39.7 |  | 1.2 |
| (+)-Pulegone |  | 10.0 |  | 41.1 |  | 0.9 |
| 1-chlorodecane |  | 60.8 |  | 42.1 |  | 0.4 |
| 1-decanol |  | 58.0 |  | 42.1 |  | 0.9 |
| menthyl acetate |  | 10.0 |  | 42.4 |  | 0.7 |
| farnesene |  | 10.0 |  | 46.8 |  | 0.5 |
| caryophyllene |  | 10.0 |  | 46.8 |  | 0.4 |
| α-Cedrene |  | 10.0 |  | 46.8 |  | 0.4 |
| α-Humulene |  | 10.0 |  | 47.8 |  | 0.5 |
| valencene |  | 10.0 |  | 48.7 |  | 0.6 |
| nerolidol |  | 10.0 |  | 49.2 |  | 0.7 |
| guaiol |  | 10.0 |  | 51.4 |  | 0.8 |
| (+)-cedrol |  | 10.0 |  | 52.0 |  | 1.0 |
| (-)-α-bisabolol |  | 10.0 |  | 53.4 |  | 0.9 |

# References

[1] S. Kitchen, A. Edge, R. Smith, J. Boschmans, M. Allsworth, B. Boyle, P. Thomas, S. Fowler, S. Siddiqui, M.v.d. Schee, Breathe Free: open source development of a breath sampler by a consortium of breath researchers, in: B.F.T.O.S.B.S. Consortium (Ed.) <http://www.breathe-free.org/>, 2015.
